# Supplementary material for: Unveiling the Bioactive Potential of the Invasive Jellyfish Phyllorhiza punctata Through Integrative Transcriptomic and Proteomic Analyses
Source: Biomolecules. 2025 Aug 4;15(8):1121. doi: 10.3390/biom15081121 (PMC12383608; doi:10.3390/biom15081121)
Supplement: Supplementary file 1 [file biomolecules-15-01121-s001.zip › Suplementary Material/FileS3/PEPFOLD4/XP_065053682_13593/clusters.html]

 PEP-FOLD clusters


Download archive of all models  here   

|  |  |  |
| --- | --- | --- |
| # | file | sOPEP |
| # cluster 1 - Size: 73 |  |  |
|  | XP\_065053682\_13593-model1 | -2.70763 |
|  | XP\_065053682\_13593-model1.1 | -2.70763 |
|  | XP\_065053682\_13593-model1.2 | -2.70095 |
|  | XP\_065053682\_13593-model1.3 | -2.67255 |
|  | XP\_065053682\_13593-model1.4 | -2.67011 |
|  | XP\_065053682\_13593-model1.5 | -2.67011 |
|  | XP\_065053682\_13593-model1.6 | -2.67011 |
|  | XP\_065053682\_13593-model1.7 | -2.67011 |
|  | XP\_065053682\_13593-model1.8 | -2.67011 |
|  | XP\_065053682\_13593-model1.9 | -2.67011 |
|  | XP\_065053682\_13593-model1.10 | -2.67011 |
|  | XP\_065053682\_13593-model1.11 | -2.67011 |
|  | XP\_065053682\_13593-model1.12 | -2.67011 |
|  | XP\_065053682\_13593-model1.13 | -2.67011 |
|  | XP\_065053682\_13593-model1.14 | -2.67011 |
|  | XP\_065053682\_13593-model1.15 | -2.67011 |
|  | XP\_065053682\_13593-model1.16 | -2.67011 |
|  | XP\_065053682\_13593-model1.17 | -2.67011 |
|  | XP\_065053682\_13593-model1.18 | -2.67011 |
|  | XP\_065053682\_13593-model1.19 | -2.67011 |
|  | XP\_065053682\_13593-model1.20 | -2.67011 |
|  | XP\_065053682\_13593-model1.21 | -2.67011 |
|  | XP\_065053682\_13593-model1.22 | -2.67011 |
|  | XP\_065053682\_13593-model1.23 | -2.67011 |
|  | XP\_065053682\_13593-model1.24 | -2.67011 |
|  | XP\_065053682\_13593-model1.25 | -2.67011 |
|  | XP\_065053682\_13593-model1.26 | -2.67011 |
|  | XP\_065053682\_13593-model1.27 | -2.67011 |
|  | XP\_065053682\_13593-model1.28 | -2.67011 |
|  | XP\_065053682\_13593-model1.29 | -2.67011 |
|  | XP\_065053682\_13593-model1.30 | -2.67011 |
|  | XP\_065053682\_13593-model1.31 | -2.67011 |
|  | XP\_065053682\_13593-model1.32 | -2.67011 |
|  | XP\_065053682\_13593-model1.33 | -2.67011 |
|  | XP\_065053682\_13593-model1.34 | -2.66266 |
|  | XP\_065053682\_13593-model1.35 | -2.66266 |
|  | XP\_065053682\_13593-model1.36 | -2.66266 |
|  | XP\_065053682\_13593-model1.37 | -2.66266 |
|  | XP\_065053682\_13593-model1.38 | -2.66266 |
|  | XP\_065053682\_13593-model1.39 | -2.66266 |
|  | XP\_065053682\_13593-model1.40 | -2.66266 |
|  | XP\_065053682\_13593-model1.41 | -2.66266 |
|  | XP\_065053682\_13593-model1.42 | -2.66266 |
|  | XP\_065053682\_13593-model1.43 | -2.65738 |
|  | XP\_065053682\_13593-model1.44 | -2.65738 |
|  | XP\_065053682\_13593-model1.45 | -2.65738 |
|  | XP\_065053682\_13593-model1.46 | -2.65738 |
|  | XP\_065053682\_13593-model1.47 | -2.65738 |
|  | XP\_065053682\_13593-model1.48 | -2.65738 |
|  | XP\_065053682\_13593-model1.49 | -2.65738 |
|  | XP\_065053682\_13593-model1.50 | -2.65738 |
|  | XP\_065053682\_13593-model1.51 | -2.65139 |
|  | XP\_065053682\_13593-model1.52 | -2.65139 |
|  | XP\_065053682\_13593-model1.53 | -2.65139 |
|  | XP\_065053682\_13593-model1.54 | -2.65139 |
|  | XP\_065053682\_13593-model1.55 | -2.65139 |
|  | XP\_065053682\_13593-model1.56 | -2.65139 |
|  | XP\_065053682\_13593-model1.57 | -2.65139 |
|  | XP\_065053682\_13593-model1.58 | -2.65139 |
|  | XP\_065053682\_13593-model1.59 | -2.65139 |
|  | XP\_065053682\_13593-model1.60 | -2.65139 |
|  | XP\_065053682\_13593-model1.61 | -2.64613 |
|  | XP\_065053682\_13593-model1.62 | -2.64613 |
|  | XP\_065053682\_13593-model1.63 | -2.64613 |
|  | XP\_065053682\_13593-model1.64 | -2.64613 |
|  | XP\_065053682\_13593-model1.65 | -2.64613 |
|  | XP\_065053682\_13593-model1.66 | -2.64613 |
|  | XP\_065053682\_13593-model1.67 | -2.64613 |
|  | XP\_065053682\_13593-model1.68 | -2.6435 |
|  | XP\_065053682\_13593-model1.69 | -2.63993 |
|  | XP\_065053682\_13593-model1.70 | -2.63993 |
|  | XP\_065053682\_13593-model1.71 | -2.62867 |
|  | XP\_065053682\_13593-model1.72 | -2.61483 |
| # cluster 2 - Size: 27 |  |  |
|  | XP\_065053682\_13593-model2 | -2.6702 |
|  | XP\_065053682\_13593-model2.1 | -2.6702 |
|  | XP\_065053682\_13593-model2.2 | -2.6702 |
|  | XP\_065053682\_13593-model2.3 | -2.6702 |
|  | XP\_065053682\_13593-model2.4 | -2.6702 |
|  | XP\_065053682\_13593-model2.5 | -2.6702 |
|  | XP\_065053682\_13593-model2.6 | -2.6702 |
|  | XP\_065053682\_13593-model2.7 | -2.6702 |
|  | XP\_065053682\_13593-model2.8 | -2.6702 |
|  | XP\_065053682\_13593-model2.9 | -2.6702 |
|  | XP\_065053682\_13593-model2.10 | -2.6702 |
|  | XP\_065053682\_13593-model2.11 | -2.6702 |
|  | XP\_065053682\_13593-model2.12 | -2.6702 |
|  | XP\_065053682\_13593-model2.13 | -2.6702 |
|  | XP\_065053682\_13593-model2.14 | -2.66776 |
|  | XP\_065053682\_13593-model2.15 | -2.66776 |
|  | XP\_065053682\_13593-model2.16 | -2.66776 |
|  | XP\_065053682\_13593-model2.17 | -2.6619 |
|  | XP\_065053682\_13593-model2.18 | -2.6619 |
|  | XP\_065053682\_13593-model2.19 | -2.6619 |
|  | XP\_065053682\_13593-model2.20 | -2.6619 |
|  | XP\_065053682\_13593-model2.21 | -2.6619 |
|  | XP\_065053682\_13593-model2.22 | -2.6619 |
|  | XP\_065053682\_13593-model2.23 | -2.65123 |
|  | XP\_065053682\_13593-model2.24 | -2.65123 |
|  | XP\_065053682\_13593-model2.25 | -2.6475 |
|  | XP\_065053682\_13593-model2.26 | -2.63938 |
|  |  |  |
